# Turning T cells on: epigenetically enhanced expression of effector T-cell costimulatory molecules on irradiated human tumor cells

**DOI:** 10.1186/2051-1426-1-17

**Published:** 2013-09-23

**Authors:** Anita Kumari, Ercan Cacan, Susanna F Greer, Charlie Garnett-Benson

**Affiliations:** 1 Department of Biology, Center for Inflammation, Infection and Immunity, Georgia State University, 161 Jesse Hill Jr. Dr, Atlanta, GA, USA

**Keywords:** External beam radiation, Immunogenic modulation, CTLs, Epigenetic, Effector co-stimulation

## Abstract

**Background:**

Sub-lethal doses of radiation can alter the phenotype of target tissue by modulating gene expression and making tumor cells more susceptible to T-cell-mediated immune attack. We have previously shown that sub-lethal tumor cell irradiation enhances killing of colorectal carcinoma cells by tumor-specific cytotoxic T cells by unknown mechanisms. Recent data from our lab indicates that irradiation of tumor cells results in the upregulation of OX40L and 41BBL, and that T cells incubated with irradiated tumor cells displayed improved CTL survival, activation and effector activity. The objective of this current study was to determine the mechanism of enhanced OX40L and 41BBL expression in human colorectal tumor cells.

**Methods:**

Two colorectal carcinoma cell lines, HCT116 and SW620, were examined for changes in the expression of 41BBL and OX40L in response to inhibition of histone deacetylases (using TSA) and DNA methyltransferases (using 5-Aza-2′-deoxycytidine) to evaluate if epigenetic mechanisms of gene expression can modulate these genes. Tumor cells were treated with radiation, TSA, or 5-Aza-dC, and subsequently evaluated for changes in gene expression using RT-qPCR and flow cytometry. Moreover, we assessed levels of histone acetylation at the 41BBL promoter using chromatin immunoprecipitation assays in irradiated HCT116 cells.

**Results:**

Our data indicate that expression of 41BBL and OX40L can indeed be epigenetically regulated, as inhibition of histone deacetylases and of DNA methyltransferases results in increased OX40L and 41BBL mRNA and protein expression. Treatment of tumor cells with TSA enhanced the expression of these genes more than treatment with 5-Aza-dC, and co-incubation of T cells with TSA-treated tumor cells enhanced T-cell survival and activation, similar to radiation. Furthermore, chromatin immunoprecipitation experiments revealed significantly increased histone H3 acetylation of 41BBL promoters specifically following irradiation.

**Conclusions:**

Full understanding of specific mechanisms of immunogenic modulation (altered expression of immune relevant genes) of irradiated tumor cells will be required to determine how to best utilize radiation as a tool to enhance cancer immunotherapy approaches. Overall, our results suggest that radiation can be used to make human tumors more immunogenic through epigenetic modulation of genes stimulatory to effector T-cells.

## Background

Previous reports by us and others demonstrate that sub-lethal doses of radiation alter the expression of genes within tumor cells [1-3]. Furthermore, it has been directly demonstrated that tumor irradiation, as well as treatment with some chemotherapy drugs, results in increased susceptibility to killing of tumor cells by cytotoxic T cells (CTLs) [1,4,5]. Notably, many genes that are important for T-cell anti-tumor effector activity are up-regulated following treatment with sub-lethal doses of radiation [2,4,6]. However, the mechanisms of radiation-mediated changes in the expression of such immune stimulatory genes are poorly understood.

It is clear that human cells respond to DNA-damage from ionizing radiation (IR) by inducing the expression of a number of genes at the transcriptional level [4,7,8]. Induction of altered gene expression can be due to direct cellular radiation effects or to radiation-induced changes in cellular milieu. Direct cellular effects appear to be regulated through parallel signaling pathways that originate from the nucleus following DNA damage, as well as signaling pathways that originate in the cytoplasm via reactive oxygen species production [7,9]. These pathways induce NF-kB activation and nuclear translocation [10,11]. As would be expected, DNA damage by IR can induce cellular stress responses, which result in activation of DNA damage repair pathways and apoptotic pathways [6,12]. Interestingly, regulation of the expression of a variety of genes, not related to known or typical DNA repair or apoptotic pathways, also occurs [2,13,14]. Indeed, we previously examined 23 human carcinoma cell lines for their phenotypic response to sub-lethal doses of IR [4], and found that RT increased the expression of several genes commonly down-regulated by tumors to escape immune recognition and elimination [15-20], including Fas (CD95), Intercellular adhesion molecule-1 (ICAM-1/CD54), tumor associated antigens (TAA) and major histocompatibility (MHC)-Class I. Most recently we found that radiation enhances the expression of OX40 ligand (OX40L/TNFSF4/CD134L/CD252) and 41BB ligand (41BBL/TNFSF9/CD137L), important co-stimulators of effector CTLs on tumor cells (submitted manuscript).

To elicit an effective immune response against tumors, T cells need to recognize tumor antigens presented by MHC in conjunction with appropriate co-stimulation [21,22]. In the absence of proper co-stimulation, these anti-tumor T cells become anergic. Proteins such as 41BBL and OX40L represent important co-stimulators of effector CTL activity [23-26], and we have seen sub-lethal doses of radiation increase their expression in human tumor cells; however, the mechanisms regulating radiation-enhanced modulation of the expression of these two genes remain unclear. OX40 (TNFRSF4/CD134) was originally characterized as a transiently expressed co-stimulatory molecule regulating CD4 and CD8 immunity [27], and signaling through OX40 promotes T-cell survival and expansion [28]. 41BBL co-stimulation of 41BB (TNFRSF9/CD137) on tumor-specific T cells is important for T-cell proliferation [29,30], cytokine production, and activation [31]. Engagement of OX40 and 41BB by agonist antibodies increases immunity against tumors, resulting in long-term survival [32] in a number of murine tumor models [33,34]. Recent evidence indicates that expression of 41BBL is transcriptionally activated by HDAC inhibitors in leukemia cell lines [35], and that HDAC11 plays an essential role in regulating OX40L expression [36]. Interestingly, radiation has been shown to inhibit the expression of HDAC1 and HDAC2 [37], and we have seen enhanced cytolysis by T-cells following tumor irradiation. Thus, epigenetic mechanisms may be at work during radiation-enhanced susceptibility to T-cell killing.

Epigenetic changes such as histone modifications and DNA methylation play important roles in regulating gene expression. DNA methyltransferase enzyme (DNMT1) adds methyl group to cytosine residues [38]. DNA hypermethylation of CpG dinucleotides accumulates in promoter regions of genes and contributes to their loss through epigenetic silencing. Promoter hypermethylation and genome-wide hypomethylation alters genes expression in colorectal cancer [39]. It has been found that genes having hypermethylation also exhibit altered acetylation and methylation of histones [40]. Histone acetylation via histone acetyltransferases (HATs) is another major epigenetic mechanism controlling gene expression [41-43]. Gains in histone acetylation neutralize the positive charge on lysine residues and contribute to disrupted nucleosome structure, allowing unfolding of DNA, increased transcription factor access and enhanced gene expression [44-46]. HDACs remove acetyl groups from histones and return DNA to a less accessible conformation, thereby decreasing transcription [47-49]. Alterations in HAT and HDAC activity have been identified in many human cancers [50,51]. HDAC inhibitors (HDACi) therefore promote hyperacetylation of histones, which in turn leads to chromatin relaxation and selective expression of genes.

The roles of DNA methylation and histone acetylation in the expression of OX40L and 41BBL in response to radiation have not been investigated. Hence, we designed the present study to test the hypothesis that irradiation leads to increased expression of OX40L and 41BBL in colorectal tumor cells via epigenetic regulation. We measured the expression of effector CTL co-stimulatory molecules OX40L and 41BBL on human colorectal tumor cells lines after treatment with trichostatin (TSA) and 5-Aza-2′-deoxycytidine (5-Aza-dC). Ours is the first study to report that *a*) OX40L and 41BBL expression increases in CRC cells when DNMTs are inhibited, *b*) expression of OX40L and 41BBL increases in human CRC cells when HDACs are inhibited, *c*) HDAC inhibition in CRC cells can increase the activation and survival of T cells, and *d*) radiation treatment of tumor cells results in epigenetic modification of the histones in the promoter of the costimulatory gene 41BBL. The use of ionizing radiation to specifically enhance cancer immunotherapy (CIT) strategies through epigenetic modulation of genes stimulatory to CTLs will have a broad impact on cancer therapy approaches and will extend the use of radiation into new directions.

## Methods

### Cell lines

Human colorectal carcinoma cell lines HCT116 cells were obtained from the laboratory of tumor immunology and biology, NCI, NIH. The cell line SW620 was kindly provided by Zhi-Ren Liu [52] from Georgia State University, Department of Biology. All cells were cultured as recommended by ATCC and tested periodically to ensure absence of Mycoplasma. Cells were incubated at 37°C incubator with 5% CO_2_.

### Reagents

5-Aza-2′-deoxycytidine (5-Aza-dC) and Trichostatin A (TSA) were purchased from Sigma-Aldrich (St. Louis, MO). Antibodies recognizing histone H3 and acetylated histone H3 were from Millipore (Lake Placid, NY). Cell viability following treatment was determined using Trypan blue dye exclusion on a Countess automated cell counter (Life Technologies).

### Irradiation

A RS-2000 biological X-ray irradiator (Rad source technology, Suwanee, GA) was used to irradiate tumor cells. Cells were irradiated at a dose rate of 2Gy/min by setting irradiator voltage and current at 160 kV and 25 mA, respectively. Cells were maintained in suspension and kept on ice during irradiation. Immediately after irradiation, the culture media was replaced with the fresh media.

### Quantitative real time PCR

Cells were plated and treated with 5AZA-dC (20 uM), TSA or 10Gy radiation. Untreated control cells were cultured with the equivalent amount of DMSO present in drug treated samples. Adherent and viable cells were collected and RNA was extracted from tumor cells using RNeasy mini kit (Qiagen Inc. Valencia, CA) according to manufacturer’s instructions. Purified RNA was DNase-treated by Rnase-free DNase (Qiagen Inc. Valencia, CA) following manufacturer’s instructions. Expression of OX40L and 41BBL mRNA was determined using real time RT-PCR. cDNA was synthesized using 500 ng of mRNA. Amplification of cDNA was done using Dynamo cDNA synthesis kit (Finnzymes. Vantaa, Finland). Quantitative RT-PCR was conducted using TaqMan gene expression assay (Applied Biosystems; OX40L; Hs00967195, 41BBL; Hs00169409, and HPRT; Hs99999909) according to manufacturer’s protocol. PCR thermal cycling condition was 50°C for 2 min, 95°C for 10 min, 40 cycles of 95°C for 15 sec and 60°C for 1 min in a total volume of 20 μl/reaction. Data were collected using a 7500 Real Time PCR System. All samples were run in duplicate. Hypoxanthine phosphoribosyltransferase (HPRT) was used as an endogenous house-keeping control gene and samples were normalized to expression of this gene, which was unchanged by treatment. Data were analyzed using the comparative Ct method [53].

### Flow cytometry

Cells were stained with primary labeled mAb CD137L (41BBL)-PE, and CD252 (OX40L)-PE purchased from BioLegend (San Diego, CA). Surface staining was done in cell staining buffer for 30 min on ice. Flow cytometry data were acquired on BD Fortessa and analyzed with FlowJo software (TreeStar, version 9.6). Isotype control was kept less than 5% in all the samples. Expression was considered increased if the absolute percent positive population increased by 10% or greater.

### Chromatin Immunoprecipitation (ChIP) Assay

ChIP assays were performed as previously described [54]. Briefly, 48 h after irradiation (10Gy) and TSA (500 nM) treatment cells were seeded at a density of 2.0 × 10^6^ and crosslinked with 1% formaldehyde. The crosslinking reaction was stopped by the addition of 0.125 M glycine. Cell nuclei were isolated and concentrated by lysing in SDS lysis buffer (1% SDS, 10 mM EDTA, 50 mM Tris pH 8.0, plus protease inhibitors) on ice followed by flash freezing in liquid nitrogen. Cell nuclei were sonicated using a Bioruptor to generate an average of 500 bp of sheared DNA; DNA shearing was confirmed by subjecting lysates to agarose gel electrophoresis. Sonicated lysates were then precleared with salmon-sperm/agarose beads (Upstate) and 5% of the total lysate was stored as input for normalization. Half of the remaining lysate was immunoprecipitated with control antibody, and the other half was immunoprecipitated with 5 μg of indicated antibody overnight at 4°C. Following an additional two hour immunoprecipitation with salmon-sperm/agarose beads, all samples were washed with each of the following buffers: low salt buffer (0.1% SDS, 1% Triton X-100, 2 mM EDTA, 20 mM Tris pH 8.0, 150 mM NaCl), high salt buffer (0.1% SDS, 1% Triton X-100, 2 mM EDTA, 20 mM Tris pH 8.0, 500 mM NaCl), LiCl buffer (0.25 M LiCl, 1% NP40, 1% DOC, 1 mM EDTA, 10 mM Tris pH 8.0), and 1xTE buffer. DNA was eluted with SDS elution buffer (1% SDS, 0.1 M NaHCO_3_) and then cross-links were reversed overnight with 5 M NaCl at 65°C and immunoprecipitated DNA was isolated using phenol:chloroform:isopropanol mix (Invitrogen) as per the manufacturer’s instructions. Isolated DNA was quantified by real time PCR on an ABI prism 7900 (Applied Biosystems, Foster City, CA) using the following primers and probe for 4-1BBL: forward, 5’- GCA CGC ATA GAC ATA AAT TGG C-3’, reverse, 5’-TCT GTG TCT CCC CGT TAA C -3’ and probe, 5’-TCC ACC CAC TGC AGA GGC AAT CAA-3’; for GAPDH: forward, 5’-AAT GAA TGG GCA GCC GTT A-3’, reverse, 5’-TAG CCT CGC TCC ACC TGA CT-3’ and probe, 5’-CCT GCC GGT GAC TAA CCC TGC GCT CCT-3’; and for CIITA: forward, 5’-CAG TTG GGA TGC CAC TTC TGA-3’, reverse, 5’- TGG AGC AAC CAA GCA CCT ACT-3’ and probe, 5’-AAG CAC GTG GTG GC-3’. Values generated from real time PCR reactions were calculated based on standard curves generated, were run in triplicate reactions and were analyzed using the SDS 2.0 program.

### Generation TAA-specific cytotoxic T-lymphocytes

PBMCs from HLA-A2+ donors were purchased from Hemacare (Van Nuys, CA) for the generation of antigen specific CTLs as described elsewhere [4,55,56]. These leukapheresis samples, derived from HLA-A2+ patients, were obtained from Hemacare with appropriate informed consent. The use of these de-identified and commercially purchased tissues is under a human investigation protocol approved by the GSU IRB (exempt approval #H13305). Briefly, PMBCs were allowed to adhere to T150 flask for 2 hr in AIM-V media. After 2 hr, non-adherent cells were removed for lymphocyte isolation. Adherent cells were cultured for seven days in the presence of 100 ng/ml of human granulocyte colony stimulating factor (GM-CSF) and 20 ng/ml of IL-4 (Miltenyi Biotec, Inc. Auburn, CA) in AIM-V media and 500 ng/ml CD40L (Millipore corporation, Temecula, CA) was added on day five to mature the DCs. On day seven DCs were collected and pulsed with 40 μg/mL of HLA-A2 binding CEA peptide (YLSGANLNL (CAP-1; [56],) peptide for 4 hr in a 37°C 5% CO_2_ incubator. Unused DCs were frozen and stored in liquid nitrogen for subsequent restimulations. DCs loaded with peptide were subsequently irradiated with 50Gy. Immunomagnetic beads (Miltenyi Biotec Inc. Auburn, CA) were used to isolate CD8+ T cells from the non-adherent cells, following manufacturer instructions. Subsequently, isolated CD8+ T cells were co-cultured with peptide pulsed DCs. IL-7 (Millipore, Temecula, CA) at 10 ng/ml and IL2 (Millipore, Temecula, CA) at 30U/ml were added to each well on the first and third day, respectively. T-cells were restimulated in this manner weekly using mature autologous DCs. Restimulated T cells were isolated over ficoll on day seven of culture, and used in a T-cell activation and survival assays.

### T-cell activation and survival assay

1 × 10^3^ colorectal tumor cells were irradiated or treated with TSA and plated in 96-well plate for 48 hr. 1 × 10^4^ human CEA specific CD8+ T cells were subsequently added and co-cultured with the colorectal tumor cells for 48 hr. The percent of CD8+ T cells expressing CD69 or CD25 was measured by flow-cytometry. In parallel experiments, 7AAD was used to measure T-cell death. Flow cytometry data were acquired on BD Fortessa and analyzed with FlowJo software (TreeStar, version 9.6). The live cells population was gated on the FSC and SSC scatter plots for analysis of surface proteins. No live cells gate was used for cell death analysis samples. T cell stimulation for 24 h using a (1×) cocktail of PMA and ionomycin (eBioscience) was used as a positive control for activation of TAA-specific T-cells.

### Statistical analysis

Statistical differences between groups were calculated using un-paired two-tailed student T-test and calculated at 95% confidence using Graphpad by Prism. P-values less that 0.05 were considered statistically significant.

## Results

### OX40L and 41BBL transcripts increase when DNMTs and HDACs are inhibited

**Figure 1:**
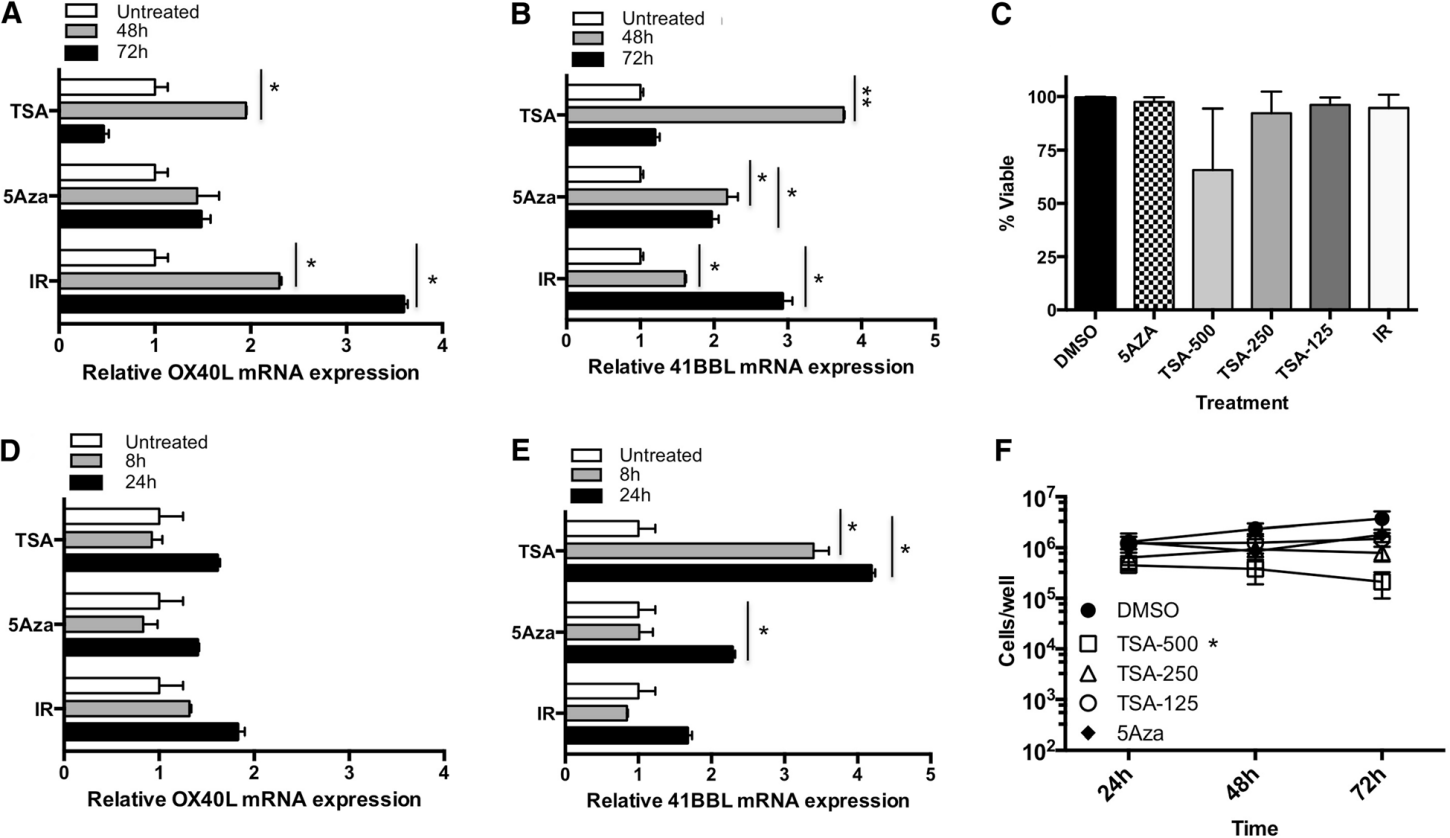
**5AZA and TSA up**-**regulate the expression of OX40L and 41BBL mRNA in HCT116 cells. (A)** OX40L, and **(B)** 41BBL mRNA level was quantified using qPCR as described in Methods. Cells were plated and treated with 5AZA-dC (20 uM), TSA (500 nM) or 10Gy radiation (IR). Adherent cell were collected after 48 (gray bar) and 72 hr (black bar) and mRNA values were compared to the level of gene expression see in untreated control samples (white bar), which was set to 1. Values represent mean ± SEM of technical replicates. *Experiments were repeated at least three times with similar results*. **(C)** Cells were plated and untreated (DMSO) or treated with 5AZA-dC (20 uM), TSA (500 nM, 250 nM or 125 nM) or 10Gy radiation (IR). Both floating and adherent cells were collected after 48 h of treatment and tumor cell viability was determining using trypan blue dye exclusion. Values represent mean ± SEM of three independent experiments. **(D)** OX40L and **(E)** 41BBL mRNA level was quantified. Adherent cell were collected after 8 (gray bar) and 24 hr (black bar) and mRNA values were compared to the level of gene expression in untreated control samples (white bar). Values represent mean ± SEM of technical replicates. *Experiments were repeated at least three times with similar results*. **(F)** Cells were plated and untreated (DMSO) or treated with 5AZA-dC (20 uM), TSA (500 nM, 250 nM or 125 nM) or 10Gy radiation (IR). Adherent cells were collected after 24, 48, and 72 h of treatment and live tumor cell number was determining using trypan blue dye exclusion. Values represent mean ± SEM of three independent experiments. Significant P-value shown in the indicated groups was determined at 48 h. *indicates P value of <0.05, **indicates P value of <0.001.

Exposure of human carcinoma cell lines to sub-lethal radiation results in enhanced susceptibility to lysis by tumor specific cytotoxic T cells (CTLs) [4,5], and co-stimulatory proteins such as 41BBL and OX40L represent important regulators of effector CTL activity [22,26]. These ligands for OX40 (OX40L/CD134L) and 41BB (41BBL/CD137L) are normally expressed on antigen presenting cells and activated endothelial cells. However, we have recently demonstrated expression of both proteins on tumor cells following treatment with radiation (submitted manuscript). Others have reported that changes in DNA methylation can upregulate the expression of costimulatory proteins in human tumor cells [57,58]. The dynamics of the induction of DNA methylation in irradiated tissue is currently unknown, and the role of methylation in expression of co-stimulatory molecules in response to radiation has not been investigated. We began our investigation by treating cells with 5-Aza-2′-deoxycytidine (5-Aza-dC) to inhibit DNA methylation in order to determine if this would alter expression of 41BBL or OX40L in human CRC cells. 5-Aza-dC is a DNA methyltransferase inhibitor (DNMTi) and is incorporated into DNA resulting in the rapid loss of DNA methyl transferase activity [59]. The human colorectal cell line HCT116 was treated with 5-Aza-dC for 48 or 72 hr, and OX40L and 41BBL mRNA was quantified. OX40L mRNA increased 1.4-fold (Figure 1A) and 41BBL mRNA increased approximately 2-fold (Figure 1B) at both 48 and 72 hr post-treatment with 5-Aza-dC. OX40L mRNA increased over time in tumor cells treated with radiation, as there was a 2.3-fold increase at 48 hr and a 3.6-fold increase at 72 hr (Figure 1A). Radiation induced a similar increase in 41BBL transcript levels. Interestingly, this temporal increase was not observed in tumor cells treated with 5-Aza-dC as relatively equal levels of both OX40L and 41BBL mRNA were detected after 48 hr (gray bar) and 72 hr (black bar) drug treatment. Moreover, the level of OX40L mRNA in cells treated 5-Aza-dC never exceeded those observed 72 h post-IR.

HDACs enzymes remove acetyl groups from histones and suppress gene transcription. Recent studies have shown that HDAC inhibitors also have immune-modulatory properties, such as increasing expression of HLA-DR, ICAM-1 and B7-2 in acute myeloid leukemia cell lines [60]. We next asked if inhibition of HDACs would result in increased expression of OX40L and 41BBL similar to the increase seen in radiation-treated cells. For these experiments we used Trichostatin A (TSA), an inhibitor of the class I and class II family of HDAC enzymes, and evaluated OX40L and 41BBL mRNA expression. HCT116 cells treated with TSA for 48 hr (gray bar) contained more OX40L (Figure 1A) and 41BBL mRNA (Figure 1B) as compared to cells treated with 5-Aza-dC for 48 or 72 hr. Messenger RNA levels decreased after 72 hr (gray bar) of TSA treatment; we note that these cells were sensitive to TSA toxicity and began dying after 48 hr TSA treatment though this loss of viability did not reach significance (Figure 1C). It is likely that mRNA expression at 48 and 72 h is not representative of early radiation events. As changes in promoter activation are often an early event we next evaluated cells at 8 and 24 h post-treatment. We found no significant increase in OX40L mRNA. Surprisingly, while radiation did not induce a significant increase in 41BBL RNA at 8 or 24 h, TSA did at both time points (Figure 1). Indeed the increase in 41BBL mRNA at 24 h (4-fold) exceeded levels observed after 48 h treatment (Figure 1B). 5-Aza-dC began to increase 41BBL as early as 24 h after treatment by slightly greater that 2-fold (Figure 1E) and this increase was maintained during 48 and 72 h treatment (Figure 1B). However, both radiation and TSA induced more 41BBL mRNA than 5-Aza-dC at their respective times of maximum induction. Overall, inhibition of both HDACs and DNMTs increased the levels of OX40L and 41BBL mRNA in HCT116 cells.

**Figure 2:**
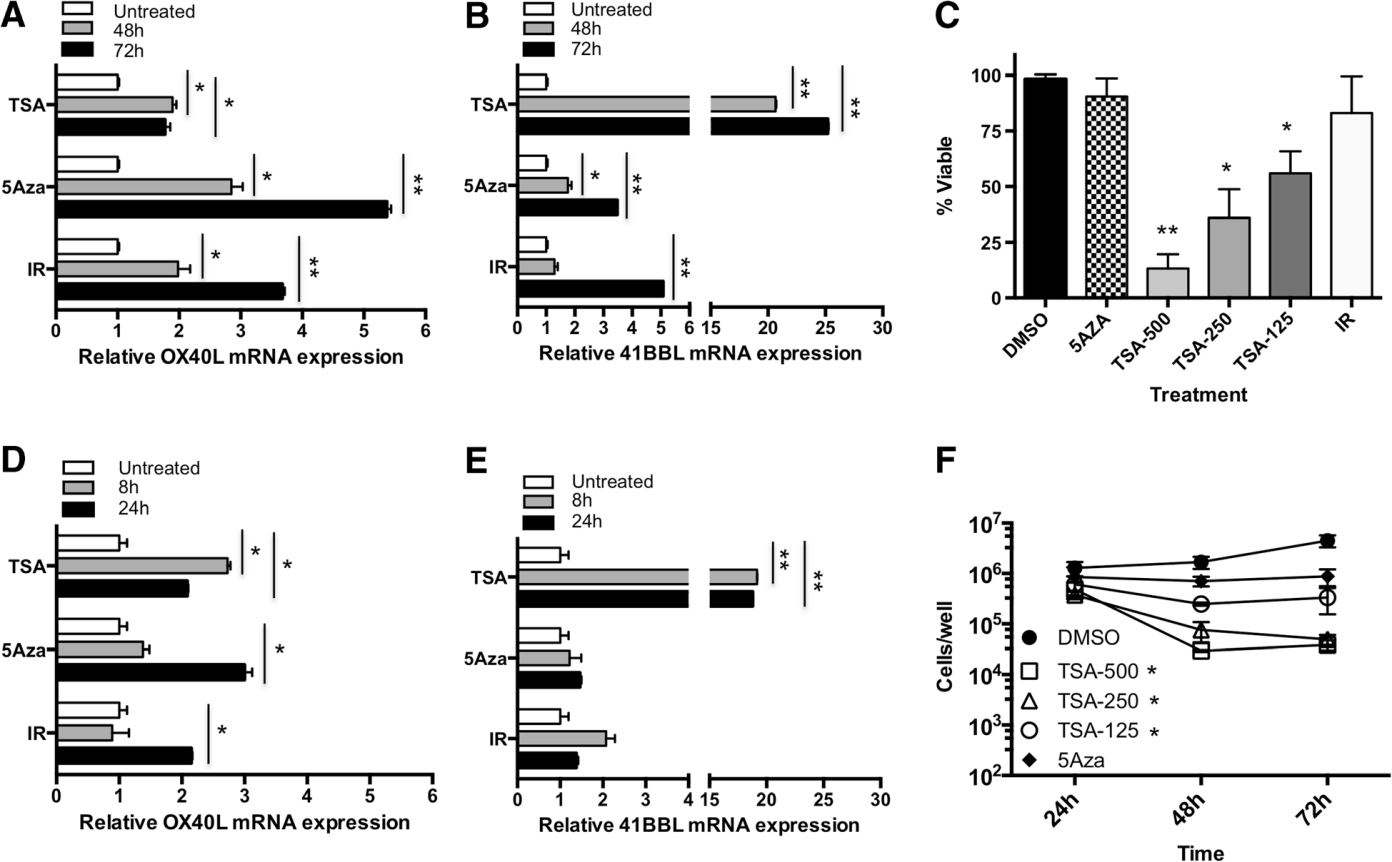
**5AZA and TSA up**-**regulate the expression of OX40L and 41BBL mRNA in SW620 cells. (A)** OX40L, and **(B)** 41BBL mRNA was quantified in SW620 cells using qPCR. Cells were plated and treated with 5AZA-dC (20 uM), TSA (250 nM) or 10Gy radiation (IR). Adherent cell were collected after 48 (gray bar) and 72 hr (black bar) and mRNA values were compared to the level of gene expression see in untreated control samples (white bar), which was set to 1. Values represent mean ± SEM of technical replicates. *Experiments were repeated at least three times with similar results*. **(C)** Cells were plated and untreated (DMSO) or treated with 5AZA-dC (20 uM), TSA (500 nM, 250 nM or 125 nM) or 10Gy radiation (IR). Both floating and adherent cells were collected after 48 h of treatment and tumor cell viability was determining using trypan blue dye exclusion. Values represent mean ± SEM of three independent experiments. **(D)** OX40L and **(E)** 41BBL mRNA level was quantified after 8 (gray bar) and 24 hr (black bar) and compared to the level of gene expression in untreated control samples (white bar). Values represent mean ± SEM of technical replicates. *Experiments were repeated at least three times with similar results*. **(F)** Cells were plated and left untreated (DMSO) or treated with 5AZA-dC (20 uM), TSA (500 nM, 250 nM or 125 nM) or 10Gy radiation (IR). Adherent cells were collected after 24, 48, and 72 h of treatment and live tumor cell number was determining using trypan blue dye exclusion. Values represent mean ± SEM of three independent experiments. Significant P-value shown in the indicated groups was determined at 48 h. *indicates P value of <0.05, **indicates P value of <0.001.

To determine if epigenetic regulation of these genes was a common mechanism observable in carcinoma cells, we evaluated a second human CRC cell line, SW620. Again, SW620 cells were treated with 5-Aza-dC and TSA for 48 or 72 hr and mRNA expression was measured by qRT-PCR. Overall, SW620 cells were more responsive to these treatments than HCT116 cells. 5-Aza-dC upregulated the expression of OX40L by 5.3 fold (Figure 2A) and 41BBL by 3.5 fold (Figure 2B) in SW620 cells treated for 72 hr (gray bar). HDAC inhibition by TSA robustly altered the expression of 41BBL mRNA resulting in a 25-fold increase (Figure 2B), and again resulted in a more modest upregulation of OX40L by 1.8-fold in SW620 cells treated for 72 hr (Figure 2A). Interestingly, these cells were more sensitive to TSA toxicity (Figure 2C) and displayed significantly reduced cell numbers following 48 and 72 h treatment with TSA concentrations ranging from 500 nM to 125 nM (Figure 2F). Viable cell numbers decreased with TSA treatment time and dose (Figure 2C), however, RNA was isolated and analyzed from the adherent and viable cells remaining in the culture (Figure 2F) for our experiments (Figure 2A & B). Moreover, we observed similar cell numbers remaining between the treatment groups after 24 h treatment with TSA and next evaluated changes in gene expression after 8 and 24 h treatment. Increased message for OX40L could be detected as early as 24 h in cells treated with radiation and 5-Aza-dC (Figure 2D) and was further increased after 48 and 72 h (Figure 2A). The largest increase in OX40L in response to TSA treatment in SW620 cells was detected following treatment for 8 h (2.7-fold) and was reduced slightly thereafter (2.1-fold). We also evaluated 41BBL expression after 8 and 24 h treatment. No significant change in 41BBL mRNA was observed at either of the earlier time points in cells treated with 5-Aza-dC or radiation. In contrast, a significant and robust increase in 41BBL expression could be detected after both 8 and 24 hr TSA treatment (20-fold) (Figure 2E) that was further increased after 72 hr treatment (Figure 2B). We noted that the relative level of 41BBL mRNA in untreated control cells appeared to be higher than OX40L mRNA levels in both cell lines evaluated. Overall, the largest increases in mRNA were detected for 41BBL mRNA following treatment of CRC cells with TSA. We also found that TSA induced robust mRNA changes at earlier times of treatment (8 h and 24 h) while radiation-induced changes took longer and were greatest at later times of treatment (48 h and 72 h).

Following tumor cell irradiation only adherent and proliferating cells were harvested for analysis. We have previously demonstrated that irradiated tumor cells continue to proliferate and remain viable using this method [6] (Figures 1C &2C). HCT116 cells appear to be less sensitive to TSA than SW620 cells as significantly reduced proliferation of treated HCT116 cells was detected only when the highest dose of TSA (500 nM) was used (Figure 1F). In contrast to TSA, there was very little impact of 5-Aza-dC on viability of tumor cells 48 h after treatment in either cell line (Figures 1C &2C). Though cell numbers were slightly reduced following 5-Aza-dC treatment of SW620 cells this was not significant (Figure 2F).

### Surface expression of OX40L and 41BBL protein increases when DNMTs and HDACs are inhibited

**Figure 3:**
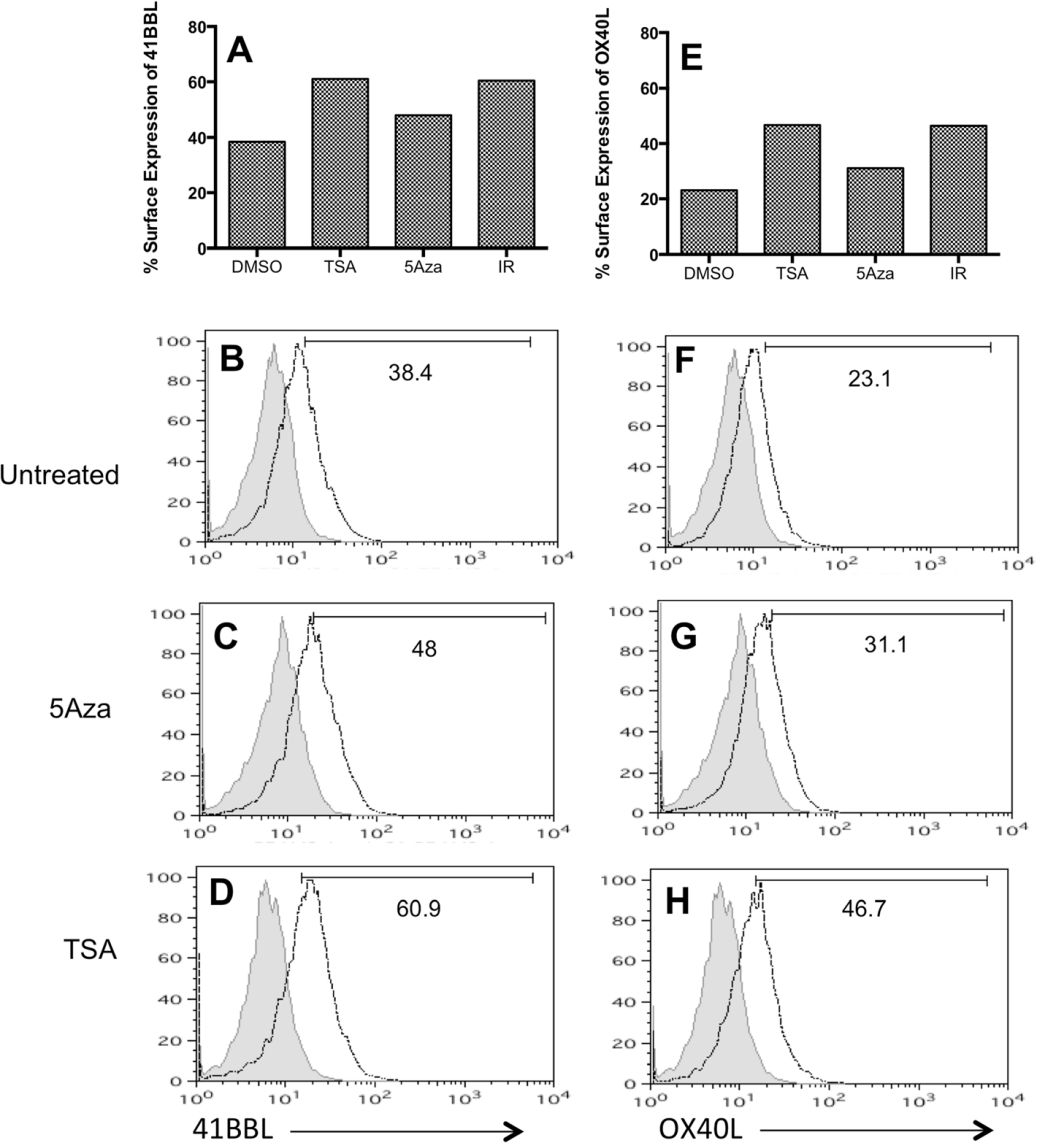
**TSA and ionizing radiation increase surface expression of co**-**stimulatory molecules in SW620 cells more than 5AZA. (A**-**D)** 41BBL, and **(E**-**H)** OX40L protein expression on the surface of SW620 cells was evaluated by flow cytometry. Cells were either untreated (DMSO), or treated with 5-Aza-dC (20 uM), TSA (125 nM) or 10Gy radiation (IR). Adherent cells were harvested 24 hr post treatment, and stained with PE-labeled antibody to human OX40L or 41BBL. Isotype control stained cells were analyzed for each treatment group individually and set to 5% positive. Isotype control staining is shown as the gray filled histogram and protein specific staining is shown as black line histogram for the FACS plot data graphed in **A** and **E**. *Experiments were repeated twice with similar results*.

The largest increase in mRNA was detected in SW620 cells treated with 5-Aza-dC (OX40L, Figure 2A) or TSA (41BBL, Figure 2B), and we wanted to determine if increased protein expression also occurred. There was no significant difference in the total cell number (Figure 2F) or the viability (data not shown) of SW620 cells following 24 h hour treatment with 125 nM TSA. As such, we evaluated surface expression of 41BBL protein by flow cytometry after 24 hr treatment with either TSA (125 nM) or 5-Aza-dC. Untreated SW620 cells expressed modest amounts of 41BBL on the surface (38.4%), and as expected radiation increased the frequency to 60.4% (Figure 3A). Treatment with 5-Aza-dC had less of an impact on protein expression and 48% of cells expressed 41BBL after treatment with the drug (Figure 3C). In contrast, TSA had a much larger impact on protein expression and, similar to radiation-induced expression, 61% of TSA-treated SW620 cells expressed 41BBL (Figure 3D) (66% in cells treated with 500 nM). Thus, relative changes in 41BBL protein expression (Figure 3A) and 41BBL mRNA quantities (Figure 2B) were similar in this cell line.

We next evaluated OX40L protein expression. SW620 tumor cells increased surface OX40L following exposure to 10Gy of radiation (IR; 46.4%), as compared to untreated cells (DMSO; 23.1%) (Figure 3E). TSA increased protein expression of OX40L to a similar magnitude (46.7%) as irradiated cells. Again, as seen with 41BBL, there was a smaller increase in surface OX40L detected (31.1%) following treatment with 5-Aza-dC. This was surprisingly low given the 3- to 5-fold increase in OX40L mRNA seen in these cells upon 5-Aza-dC treatment (Figures 2A &2D). Thus, mRNA modulation of the two genes (Figure 2) was similar to protein changes by TSA and radiation (Figure 3), but not 5-Aza-dC. Furthermore, the modulation of OX40L protein was less robust than that observed for 41BBL protein in SW620 cells (Figure 3B-3D &3F-3H).

**Figure 4:**
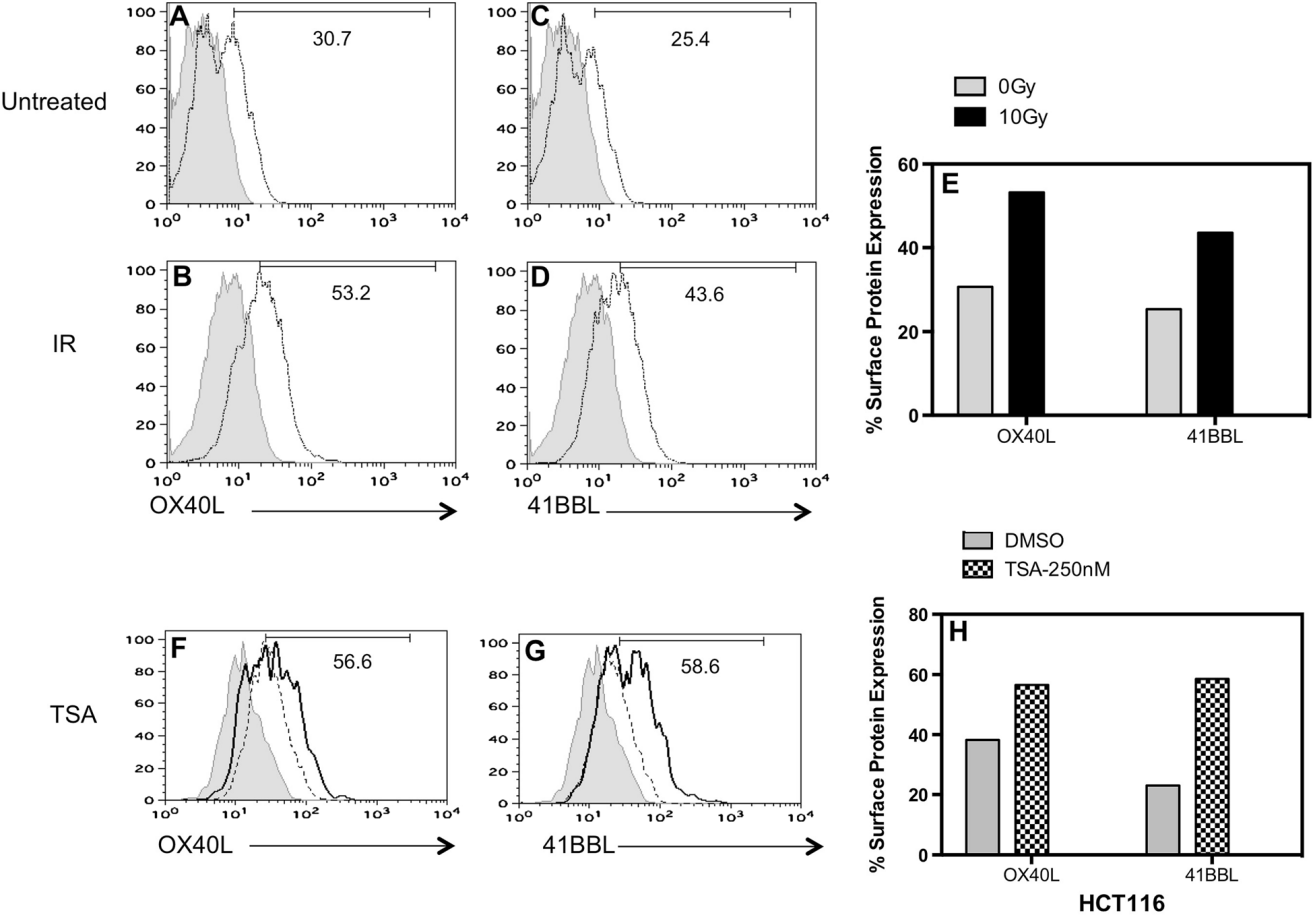
**TSA and ionizing radiation treated HCT116 cells increase surface levels of 41BBL protein more than OX40L protein. (A, B and F)** OX40L, and **(C, D and G)** 41BBL protein expression on the surface of HCT116 cells was evaluated by flow cytometry. **(E)** Cells were untreated (0Gy) or treated with 10Gy radiation (IR). **(H)** In separate experiments HCT116 cells were untreated (DMSO) or treated with either TSA (250 nM). After 48 hr, cells were collected and stained with PE-labeled antibody to either OX40L and 41BBL. Control cell staining with a non-specific isotype control antibody was less than 5% positive. Isotype control staining is shown as the gray filled histogram and protein specific staining is shown as black line histogram for the FACS plot data graphed in **E** and **H**. Dotted line histogram in F and G indicates specific protein expression in untreated cells. Values shown on histograms are for treated cells and untreated and treated values are displayed graphically in H for comparison. *Experiments were repeated three times with similar results*.

Overall, our results show that TSA-treated cells demonstrated the largest increase in protein expression, and the increase was at least as good as that observed following treatment with radiation (Figure 3). As such, we focused our subsequent experiments on the impact of TSA HDAC inhibition on co-stimulatory molecule expression. Our data reveal increased expression of OX40L (53.2%) 48 hr after irradiation of HCT116 cells as compared to untreated (0Gy) cells (30.7%) (Figure 4A-B &4E). Expression of OX40L is detected on the surface of 56.6% TSA-treated HCT116 cells (Figure 4F) as compared to expression in control (DMSO) cells (38.2%). Expression of 41BBL was also enhanced too much greater levels following treatment with both IR (43.6% 10Gy) (Figure 4C-D &4E) and TSA (58.6%-250 nM TSA versus 23%-untreated) at 48 hr (Figure 4G). The relative change in 41BBL surface expression compared to untreated cells was larger that the change in OX40L following TSA treatment in HCT116 cells (Figure 4H). Elevated levels of these co-stimulatory proteins could still be detected after 3- to 4-days of TSA treatment and radiation-induced changes where greater after 72 h (data not shown). Overall, both HCT116 and SW620 cells showed a more robust increase in expression of 41BBL as compared to OX40L protein expression upon TSA treatment.

### Radiation increases histone H3 acetylation at the 41BBL promoter

**Figure 5:**
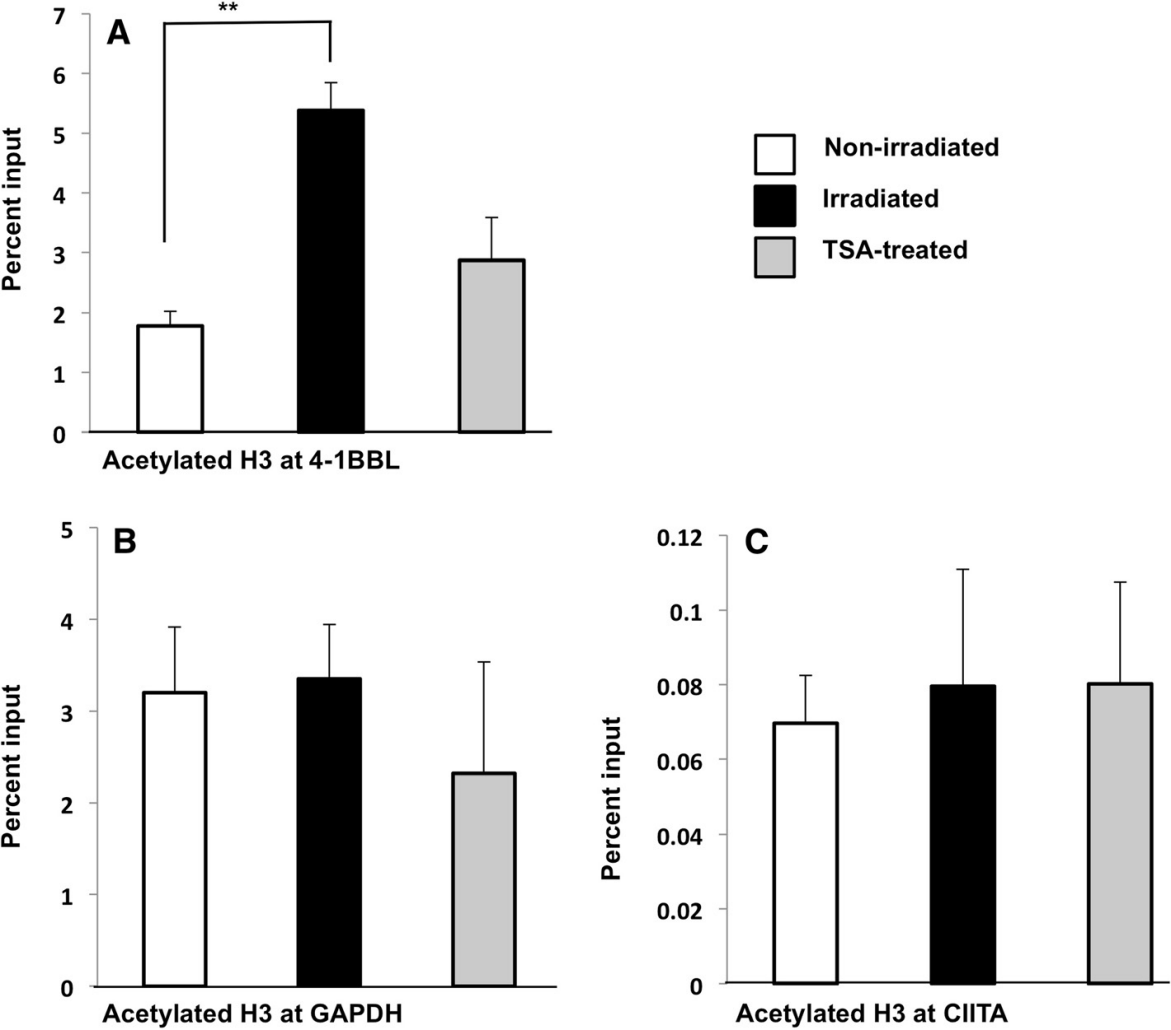
**Histone acetylation at 41BBL promoters in non**-**radiated and irradiated cells.** ChIP assays were carried out in non-irradiated, irradiated (10 Gy), and TSA-treated (500 nM) HCT116 cells. Following 48 h of TSA treatment, lysates were immunoprecipitated with control antibody or with anti-acetyl histone H3. Associated DNA was isolated and analyzed via real time PCR using primers spanning the 41BBL, GAPDH and CIITA promoters. Real-time PCR values were normalized to the total amount of promoter DNA added (input). Input values represent 5% of the total cell lysate. Values represent mean ± SEM of three independent experiments. **P < 0.005. **A**. Global levels of Histone H3 acetylation associated with the 41BBL promoter. **B**. Global levels of Histone H3 acetylation associated with the GAPDH promoter. **C**. Global levels of acetylated Histone H3 associated with the CIITA promoter.

Our data indicates that 41BBL and OX40L are epigenetically regulated and radiation increases expression of these genes in CRC cell lines. Histone acetylation facilitates transcription initiation by loosening interactions between the histones and DNA. Whereas, HDACs remove these acetyl groups from histones which reduces transcription. We observed that inhibition of HDACs by TSA increased 41BBL mRNA expression and surface protein levels in tumor cells. We observed that radiation increased 41BBL gene expression in a similar manner but was more robust at later times during treatment. As radiation has been reported to inhibit HDACs [37], we next wanted to determine if radiation could be increasing 41BBL expression by promoting increased promoter histone acetylation. To explore whether histone modifications are regulated in part by radiation, we assessed levels of histone acetylation at the 41BBL promoters using chromatin immunoprecipitation (ChIP) assays in both non-radiated and irradiated HCT116 cells. We evaluated promoter acetylation at 48 h post-IR when radiation-induced changes in mRNA levels were robust (Figure 1). TSA-treated HCT116 cells were used as a positive control for 41BBL promoter acetylation. As TSA inhibits HDAC activity, we expect to see robust increases in histone acetylation status following TSA treatment. As expected, Figure 5A shows increased acetylation at the 41BBL promoter following TSA treatment (gray bar) as compared to untreated control cells (white bar). Surprisingly, acetylated H3 histone levels were significantly higher at 41BBL promoters in irradiated cells (black bar). In contrast, similar levels of acetylated histone H3 were associated with the GAPDH promoter in both untreated and irradiated HCT116 cells (Figure 5B). Moreover, total levels of histone H3 were similar at 41BBL and GAPDH promoters revealing that there was no global change in overall histone levels (data not shown). These data indicate that radiation increases 41BBL expression by increasing histone acetylation. To determine if radiation non-specifically increases histone acetylation levels at other genes, histone H3 ChIP assays were performed on the Class II Transactivator (CIITA) promoter IV. Histone H3 acetylation levels were similar for non-irradiated, irradiated and TSA treated cells at CIITA promoter IV (Figure 5C), which suggests gene-specificity for radiation-induced 41BBL promoter acetylation, likely via HDAC inhibition.

### Treatment of CRC cells with TSA enhances T-cell survival and activation similar to co-incubation with irradiated tumor cells

**Figure 6:**
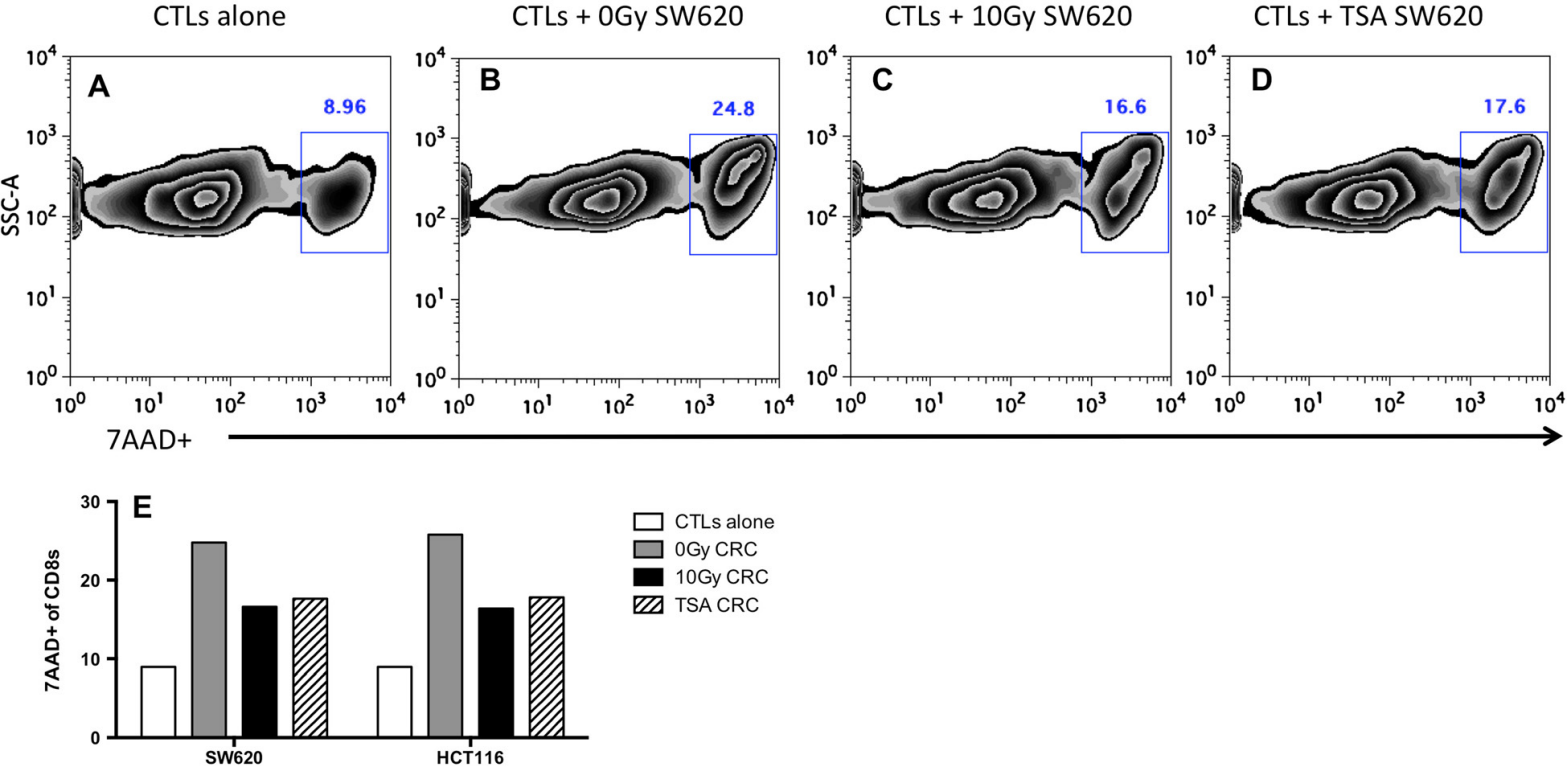
**Enhanced survival of T**-**cells co**-**incubated with CRC tumor cells treated with TSA.** SW620 and HCT116 cells were treated with DMSO, TSA (250 nM), or radiation (10Gy). After 48 hr, tumor cell were harvested and co-cultured with human CEA-specific CD8 + T-cells for another 48 hr. Cell death was also measured in CD8+ T-cells cultured alone. Data were gated first gated on CD8+ population and the percent of 7AAD-positive within the CD8+ cell population is shown in **(A**-**D)** as zebra plots. **(E)** Representative graphs showing percentage of dead CD8+ T-cells. *Experiments were repeated twice with similar results*.

To investigate the impact of HDAC inhibition in tumor cells on T-cell survival, we measured T-cell death by 7AAD staining after 48 hr co-incubation with tumor cells. 7AAD + staining determined cell death of 8.96% of CD8+ T cells incubated alone (Figure 6A). The frequency of dead CD8+ T cells increased to 24.8% following co-incubation with untreated SW620 cells (Figure 6B). Death of T-cells following interaction with tumor cells has been reported by others, and is thought to be caused by tumor expressed PDL1, FasL and/or activation induced cell death (AICD) [61-63]. Incubation of T-cells with SW620 cells, which had been treated with TSA for 48 hr, reduced the percentage of dead T cells to 17.6% (Figure 6D) similar to incubation with irradiated tumor cells (16.6%). A reduction in T-cell death (18%) was also observed when T-cells were co-incubated with TSA-treated HCT116 cells as compared to untreated tumor cells (26%) (Figure 6E). These data indicate that HDAC inhibition by TSA treatment of tumor cells increases the survival of CD8^+^ T cells following co-incubation with tumor cells.

**Figure 7:**
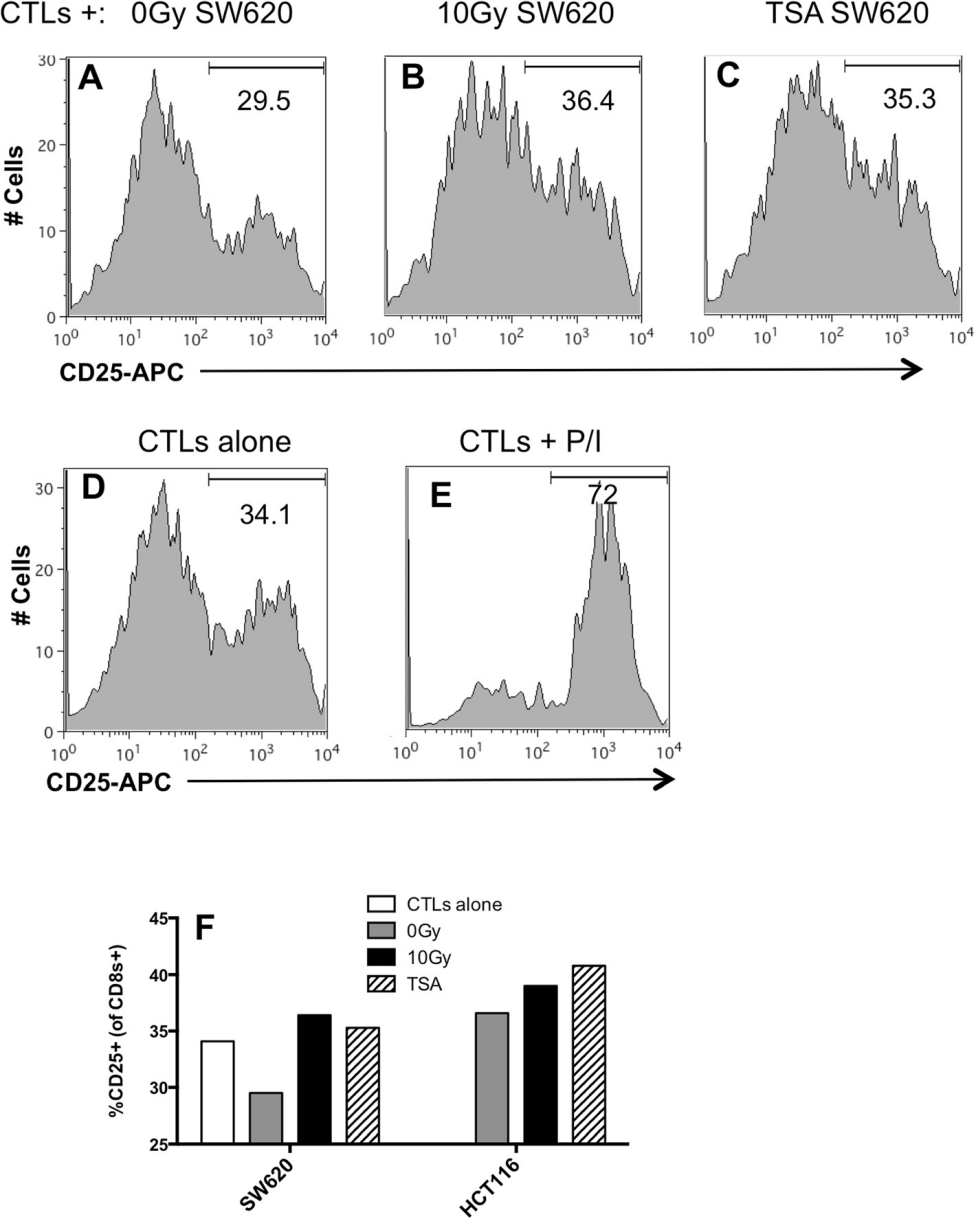
**TSA**-**treated CRC tumor cells induce enhanced activation of CD8+ T**-**cells.** SW620 cells were either **(A)** untreated, **(B)** irradiated or incubated with **(C)** 250 uM TSA for 48 hr or as previously described. Tumor cells were subsequently co-cultured with human CEA-specific CD8 + T-cells for 48 hr and the frequency of CD8 + CD25+ T-cells was measured by flow cytometry. T-cell activation was also measured in **(D)** CD8+ T-cells cultured alone or **(E)** activated with PMA and ionomycin (P/I) for 24 h. Data were gated first gated on CD8+ population and the percent of CD25+ cells within the CD8+ cell population is shown in **(A**-**E)** as histogram plots. **(F)** a summary graph of results showing percentage of CD8+ T-cells expressing CD25 in both SW620 and HCT116 cells. Experiments were repeated twice with similar results.

CD25 and CD69 are surface markers expressed on activated T cells [64]. Data from our lab supports the hypothesis that changes in the expression of tumor-expressed 41BBL and OX40L contribute to increased killing of irradiated tumor cells by CTLs (submitted manuscript). We have also observed increased expression of CD25 and CD69 on T cells following co-incubation with irradiated tumor cells compared to non-irradiated tumor cells. Lastly, we have observed increased viability of T cells cultured with irradiated tumor cells. We next determined if tumor cells treated with HDACi induced similar changes in T cell activation. Non-treated, irradiated or TSA treated tumor cells were co-cultured with CD8^+^ T cells, and after 48 hr the expression of CD25 on T cells was measured by flow cytometry. We found that 29.5% of CD8+ T cells incubated with untreated tumor cells expressed CD25 (Figure 7A), and this frequency was reduced compared to activation of T cells incubated alone (34.1%) (Figure 7D). This reduction is not surprising as reduced activity and activation of T-cells following interaction with tumor cells has been described by others [61-63]. The frequency of CD25^+^ within the CD8^+^T cell population increased following co-incubation with either radiation-treated (Figure 7B) or TSA-treated tumor cells to 35.3% (Figure 7C). In fact, the frequency of activated T cells following co-incubation with TSA-treated cells was equal to T cells not co-incubated with tumor cells (34.1%). CD25 expression in T-cells activated with PMA and ionomycin are shown as a positive control (Figure 7E). We evaluated a second CRC cell line and found that TSA-treated HCT116 cells also increased the frequency of CD8^+^CD25^+^ cells to 41%, as compared to the frequency activated in the presence of untreated HCT116 cells (36.6%) (Figure 7F). Irradiated tumor cells also increased CD25^+^ expression to 36.4% and the dynamics of T-cell activated were similar in repeat experiments. We observed a similar increase in the frequency of CD69^+^ T cells following co-incubated with TSA-treated or irradiated tumor cells (data not shown). These data suggest that T cells exposed to TSA treated tumor cells have improved activation. As a component of the IL-2 receptor, CD25 it has been linked to increased survival in studies by others and thus could be a contributor to the increased survival we observe following TSA treatment (Figure 6).

## Discussion

Modulation of costimulatory molecules such as OX40L and 41BBL appear to be particularly important for maintaining effective immune responses against self-antigens presented by tumor cells. Here, we report that costimulatory molecule promoter histones can be acetylated in colorectal tumors in response to sub-lethal radiation (Figure 5A). Most studies of radiation-induced gene expression have used large cytotoxic doses of radiation, and mechanisms of altered gene expression are much less explored in cells receiving low or sub-lethal doses of radiation. Results of this study suggest that radiation therapy may be useful to specifically modulate gene expression within tumor targets. This mechanism would be useful against radioresistant cancer cells, and could occur even in the absence of immunogenic cell death (cell death that invokes enhanced antigen processing and presentation) [65]. Full understanding of specific mechanisms of immunogenic modulation (altered expression of immune relevant genes) [66] of irradiated tumor cells will be required to determine how to best utilize radiation as a “tool” to enhance cancer immunotherapy approaches.

Dramatic changes in DNA methylation are common in cancer, and manifest primarily as global DNA hypomethylation, paralleled by local hypermethylation at gene promoters resulting in loss of gene expression [67,68]. Tumor cells down-regulate the expression of many genes needed for induction of effective anti-tumor immune activity [15,16,18,19], and DNA methylation may be one mechanism employed to accomplish this. Our studies reveal that inhibition of DNMT in tumor cells using 5-Aza-dC could induce mRNA expression of both OX40L and 41BBL on two different CRC cell lines (Figure 1 &2). Although a greater than 5-fold induction of mRNA was detected in SW620 cells treated with 5-Aza-dC, we did not observe a robust increase in protein expression upon 5-Aza-dC treatment of these cells (Figure 3). These discordant results could simply be a result of the time of evaluation post-treatment. 41BBL mRNA was maximally increased 72 hr post-treatment with 5-Aza-dC, while protein expression was evaluated after 24 h of treatment to keep cell death low at time of evaluation. Current studies are underway to determine if 5-Aza-dC can indeed upregulate protein expression at later times post-treatment.

HDAC inhibition has been shown to be involved in modulating the expression of TNF family members [69,70]. In this study we extended analysis to other TNF family members and found that both 41BBL and OX40L expression could also be modulated by inhibition of HDACs. We found that the expression of both OX40L and 41BBL was increased on the surface of tumor cells treated with TSA for 24 hr (Figure 3) or 48 hr (Figure 4). Interestingly, the impact of HDAC inhibition by TSA on 41BBL protein expression was much more robust than changes observed in the expression of OX40L protein following TSA treatment. Studies are currently underway to evaluate changes in histone acetylation at the OX40L promoter to determine how acetylation is impacted by TSA inhibition of HDACs. We also observed increased expression of co-stimulatory proteins as long as four days after TSA-treatment and irradiation. While many of the cellular stress response genes are acute response genes whose expression is altered transiently, other genes remain altered for prolonged periods of time [71-73]. As such, altered gene expression following radiation treatment that is sustained is not unexpected.

The TNF family includes numerous costimulatory molecules known to play an important role in CD8^+^ T cell activation and survival. We found that inhibition of HDACs in tumor cells resulted in enhanced T-cell survival (Figure 6) and activation (Figure 7). To our knowledge this is the first study to explore the impact of radiation-induced epigenetic changes in tumor cells on the quality of anti-tumor CTLs. We are currently investigating if, by promoting T-cell survival and activation, the altered expression of these specific genes by HDACi enhances the tumor cells’ susceptibility to T-cell-mediated immune attack in a manner similar to observations in irradiated tumor cells (submitted manuscript). Future studies seek to more fully investigate if increased signaling through CD25 is directly responsible for the increased survival of T-cells by evaluating T cells after shorter periods of co-incubation as well as investing intracellular regulators of T-cell apoptosis.

HDACs enzymes reverse the activity of HATs by removing acetyl group and thus suppressing gene transcription. In several tumors, the expression of HATs is down-regulated, whereas HDACs is upregulated [74,75]. As previously mentioned, alteration of HAT and HDAC activity has been observed in tumor cell lines. HDACi induce a potent anticancer response by inhibiting HDACs [76,77]. HDACi have various biological effects, such as inhibition of cell cycle at G1/G2 phase, induction of differentiation and apoptosis of tumor cells [78-80]. Our results reveal that radiation treatment changes the epigenetic landscape of the 41BBL gene via an increase in histone acetylation, displaying a marked increase in H3 acetylation at this specific promoter, as compared to our positive control of cells treated with the HDACi, TSA. We also observed that TSA induced robust 41BBL mRNA changes at earlier times of treatment (8 h and 24 h) while radiation-induced changes took longer and were greatest at later times of treatment (48 h and 72 h). These data, in combination with increase promoter acetylation, suggest that radiation mediated effects take longer to modulate histone acetylation events than direct modulators such as TSA. This could be related to differences in modulation of HATs versus HDAC inhibitors. Current lab efforts are pursuing the mechanism for these epigenetic changes in primary carcinoma cells; specifically, does IR treatment change the activity of HATs, HDACs or both? If HDACs are involved, specific HDAC inhibitors will be utilized to identify which HDACs suppression(s) are vital for the upregulation of 41BBL expression. Also, how long can these epigenetic changes be maintained to promote increased effector T-cell function? Finally, we note that expression of OX40L and 41BBL varied with different concentrations of drug exposure. Our focus here is to describe a novel gene regulatory mechanism by epigenetic modification in response to irradiation. However, the application of clinically relevant doses of TSA and 5-Aza-dC, which might be combined with radiation, will also require a further investigation in a broad range of tumor cells.

## Conclusions

The current study was meant to enhance our ability to design cancer immunotherapy (CIT) approaches in combination with RT. A better understanding of how IR modulates the expression of 41BBL and OX40L will allow improvement in our ability to use RT to specifically enhance CTL killing. Epigenetic mechanisms of gene expression could be an alternative therapeutic approach to enhancing these important T-cell signals. This approach is particularly relevant given the toxicities associated with using agonistic antibodies to 41BB and anti-OX40 antibodies in the clinic [28,81]. Alternate ways of triggering these signal pathways would be widely applicable in current CIT approaches. Furthermore, if radiation is shown to have a profound and consistent effect on immune stimulatory gene expression, this would provide support for using IR in conjunction with CIT strategies to specifically enhance such signals to T-cells arriving at tumor sites and optimize anti-tumor CTL responses.

## Abbreviations

5-Aza-dC: 5-Aza-2′-deoxycytidine; CEA: Carcinoembryonic antigen; CIT: Cancer immunotherapy; ChIP: Chromatin immunoprecipitation; CRC: Colorectal carcinoma; CTL: Cytotoxic T cells; DMSO: Dimethyl sulfoxide; DNMTs: DNA methyltransferases; HDACi: HDAC inhibitors; HDACs: Histone deacetylases; IR: Ionizing radiation; RT: Radiation therapy; TSA: Trichostatin A.

## Competing interests

The authors declare that they have no competing interests.

## Authors’ contributions

Conceived and designed the experiments: CGB and SFG. Performed the experiments: AK, EC and CGB. Analyzed the data: AK, EC and CGB. Contributed reagents/materials/analysis tools: CGB and SFG. Wrote the paper: CGB, AK, EC and SFG. All authors read and approved the final manuscript.

## Authors’ information

C. Garnett-Benson previously published under Garnett, C.T.
